# Multi-systemic melioidosis: a clinical, neurological, and radiological case study from Hainan Province, China

**DOI:** 10.1186/s12879-018-3569-8

**Published:** 2018-12-12

**Authors:** Wei-yuan Huang, Gang Wu, Feng Chen, Meng-meng Li, Jian-jun Li

**Affiliations:** 1 Department of Radiology, Hainan General Hospital, No. 19 Xiuhua Road, Xiuying District, Haikou, 570311 Hainan People’s Republic of China; 2 Department of Radiotherapy, Hainan General Hospital, Hainan, China; 3 Research and Education Department, Hainan General Hospital, Hainan, China

**Keywords:** Melioidosis, Burkholderia pseudomallei, Abscesses, Magnetic resonance imaging

## Abstract

**Background:**

Melioidosis is a tropical disease caused by *Burkholderia pseudomallei (B. pseudomallei).* It can infect any organ system and lead to multiple abscesses. A few studies reported that central nervous system (CNS) is also involved.

We present a diabetic patient with multi-systemic melioidosis that affected the CNS, thorax, and spleen. The aim was to study the clinical and radiological features of melioidosis and enhance understanding of the disease.

**Case presentation:**

A 38-year-old male presented with cough and expectoration mixed with blood for several days. Chest computed tomography (CT) showed a patchy opacity in his left lung, and multiple low-density lesions in his spleen. After 10 days of antibiotics treatment, his clinical symptoms improved and he was discharged from the hospital. But 8 months later, the patient experienced sudden onset of left limb weakness and seizure and was re-admitted to the hospital. Brain CT indicated a low-density lesion over the right frontal lobe, and magnetic resonance imaging (MRI) indicated a well-enhanced lobulated lesion with multiple diffusion restriction areas in the lesion. He had a neuronavigation-guided open surgery but no malignancy was found. *B. pseudomallei* was cultured from the operative samples. After 4 months of systemic and intraventricular antibiotic administration treatment, he recovered complete consciousness with left hemiparesis.

**Conclusions:**

Multi-systemic melioidosis may present atypical clinical, neurological, and radiological manifestations. It is extremely important to accurately diagnose before treatment is selected. CNS melioidosis in early stage manifests similar symptoms to malignancy or stroke. It might mislead to a false diagnose. Diffusion weighted imaging (DWI) can help in differentiate abscesses from cystic tumours.

## Background

Melioidosis is caused by *Burkholderia pseudomallei (B. pseudomallei)*, a Gram-negative bacterium (GNB) found in soil and surface water. As an infectious disease, it can cause public health problem in tropical and sub-tropical areas in Southeast Asia [1]. Hainan province in China is one of the endemic regions. People with hazardous alcohol intake, diabetes, or chronic renal disease are more likely to be infected. The latent period usually ranges from 1 to 21 days. However, latent periods delayed for months or years have also been described [2]. Melioidosis causes multiple abscesses in different organs of the body. Thoracic lesions or lymphadenopathy may present as necrotic pneumonia or necrotic nodes and mimic tuberculosis or malignancy, and the early manifestation of central nervous system (CNS) melioidosis mimics malignancy or stroke and tends to cause a diagnostic dilemma [3]. This paper reports a case of a 38-year-old patient with diabetes who recovered from multi-systemic melioidosis that involved his thorax, CNS, and spleen.

A 38-year-old male with cough and expectoration mixed with blood was hospitalised at the Department of Respiratory, Hainan General Hospital in September 2015. Through auscultation, the respiratory sounds in the left lung were weak, while a small amount of moist rale was present at the bottom of the left lung. He had 10-year smoking history and type 2 diabetes mellitus for 4 years.

**Fig. 1:**
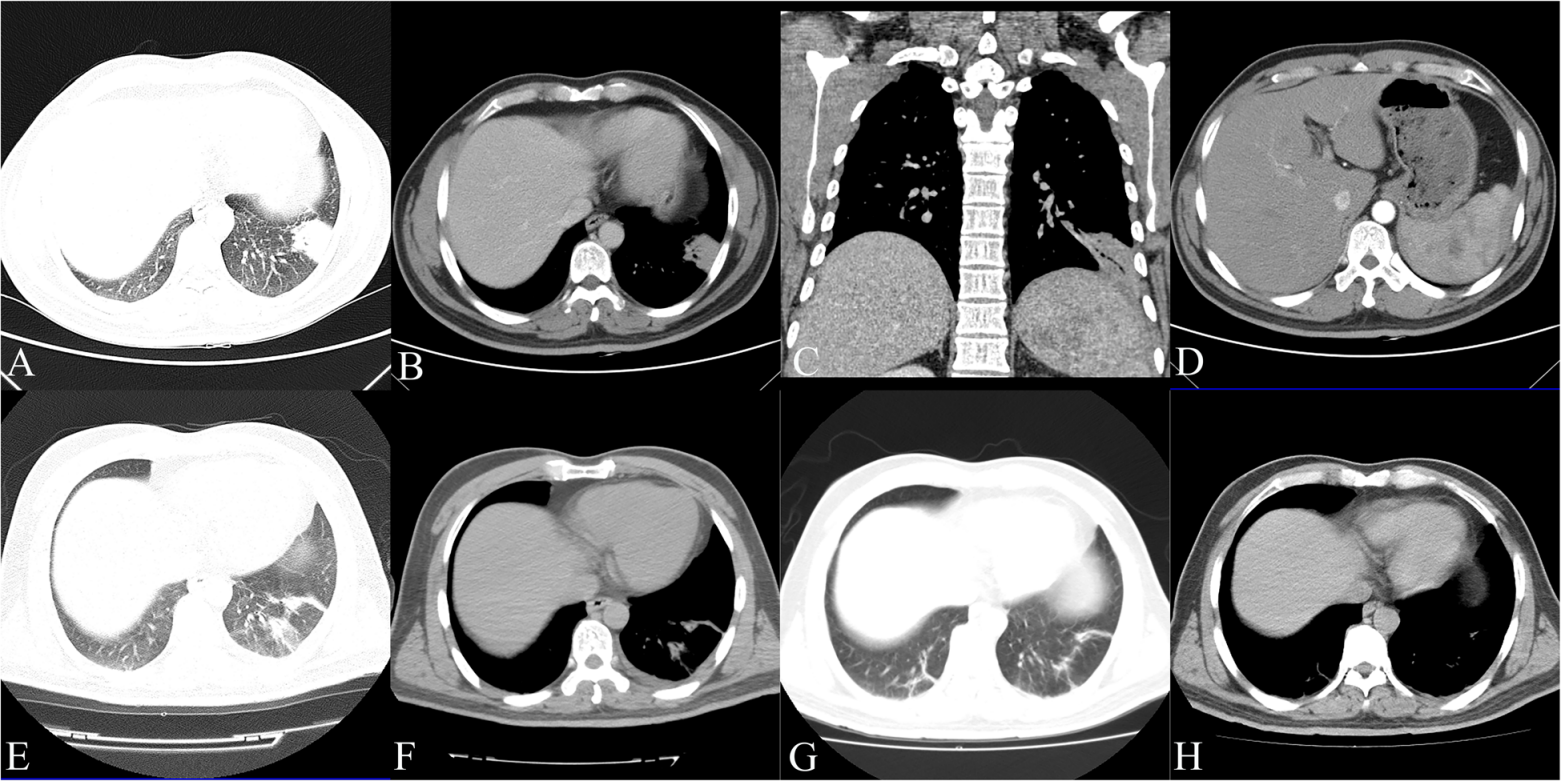
Initial chest CT (on September 11, 2015) showed a patchy opacity of 4.6 × 2.7 cm in the left lung, pleural effusion in the left chest (**a**, **b** and **c**), and multiple low-density lesions in the spleen (**d**). After a period of treatment, follow-up CT (**e** and **f**) (June 21, 2016) showed the left lung lesion had been partially, absorbed had been completely absorbed at a Aug 15, 2016, CT scan (**g** and **h**)

Routine blood tests showed an erythrocyte sedimentation rate (ESR) 35 mm, C-reactive protein (CRP) of 12.86 mg/L, and a white blood cell (WBC) count of 6.9 × 10^3^/mm^3^ with 43.8% neutrophils, normal platelets, and creatinine. The patient’s fasting plasma glucose levels was 8.69. Initial chest CT showed a patchy opacity of 4.6 × 2.7 cm in his left lung, pleural effusion appeared in the left chest, and multiple low-density lesions in the spleen (Fig. 1). Sputum smear showed a medium WBC count, less than 10 squamous epithelial cells, and a few of GNB. Blood culture was negative for bacteria. Anti-acid bacillus smear did not find acid-fast bacillus.

The pathology results examined by a percutaneous lung puncture biopsy indicated granulomatous inflammation. The patient was diagnosed with preliminary pneumonia and intravenously administered thiamphenicol to fight infection. He was also given etamsylate to stop bleeding and acarbose to reduce blood glucose. Ten days later, his condition improved and he was discharged from the hospital.

**Fig. 2:**
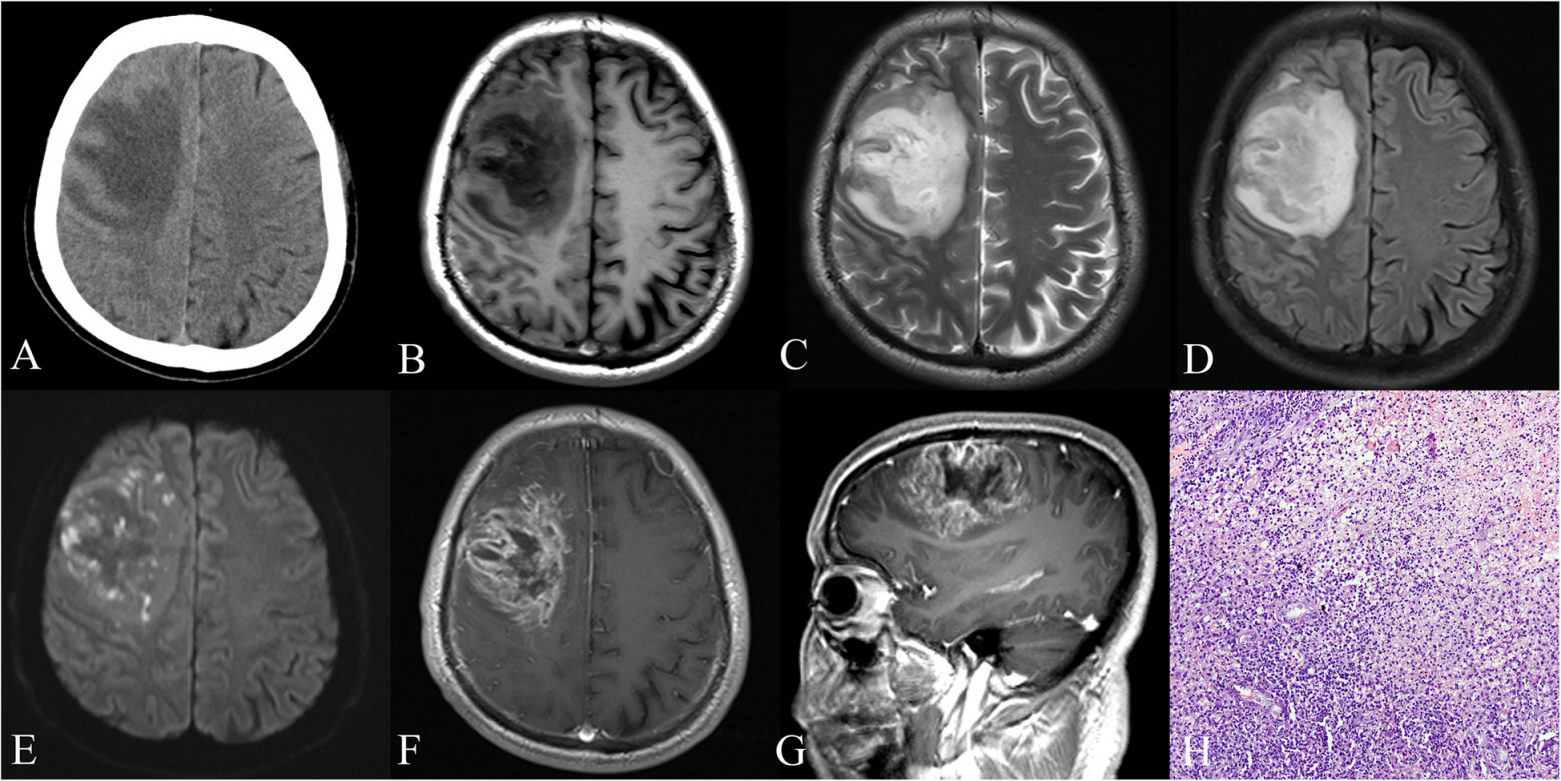
Imaging features of the case: Unenhanced CT ( **a** ) showed a low-density lesion on the left frontal lobe. Unenhanced MRI of the lesion showed hypointensity on T1 weighted-images ( **b** ) and hyperintensity on T2 weighted-images and FLAIR ( **c** and **d** ). DWI ( **e** ) showed multiple spot diffusion restriction areas in the lesion, prominently located in the centre of the lesion. Enhanced MRI of the brain ( **f** and **g** ) revealed a lobulated ring-enhancing lesion with prominent oedema and midline shift in the right frontal lobe. An intraventricular drain was placed for drainage. Histopathology ( **h** ) showed a brain abscess on the left lobe, but no evidence of malignancy. Culture of the operative specimens was positive for *Burkholderia pseudomallei* , leading to the diagnosis of melioidosis

Eight months later (June 2016), the patient experienced sudden onset of left limb weakness, hemiopia, seizure, and headache with no obvious causes. Brain CT showed a low-density lesion over the right frontal lobe (Fig. 2). He was readmitted to the hospital’s Department of Neurosurgery. On examination, his muscle strength was 0–1/5 over the left upper extremity and 1–2/5 over the left lower extremity with decreased reflex of the left extremities. His tongue extended left and the angle of his mouth on the left side was deflected. Routine blood tests showed an ESR 35 mm, CRP of 54.85 mg/L, and a WBC count of 11.14 × 10^3^/mm^3^ with 79.8% neutrophils. His fasting plasma glucose level was 8.63. Magnetic resonance imaging (MRI) of the brain showed a lobulated ring-enhancing lesion with prominent oedema and a midline shift in the right frontal lobe. Diffusion-weighed imaging (DWI) showed multiple spot diffusion restriction areas prominently located in the central part of the lesion (Fig. 2). The patient was tentatively diagnosed with malignant brain tumour (suspicious glioma) in association with left lower lobe pneumonia. Mannitol solution was injected to relieve intracranial hypertension and alleviate cerebral oedema. Oral acarbose was administered to reduce blood glucose. The patient was then prepared for surgery.

Eighteen days later, the patient presented sudden loss of consciousness, incontinence, and right pupil dilation. He had emergency craniectomy and tumour excision for midline shift caused by severe right hemisphere swelling. However, pus was found in the operative field and the lesion was considered as a brain abscess during the procedure. An intra-ventricular drain was placed for drainage. A culture of the operative specimens (pus) was identified as *B. pseudomallei* using the DL-96 bacteria identification system (Dier, Shenzhen), resulting in the diagnosis of melioidosis. Antibiotic susceptibility testing (imipenem/cilastatin, ceftazidime and sulfamethoxazole) of the operative pus showed sensitivity to imipenem/cilastatin and ceftazidime. Micro-broth dilution technique of minimum inhibitory concentration (MIC) was performed. Histopathology showed no evidence of malignancy (Fig. 2).

**Fig. 3:**
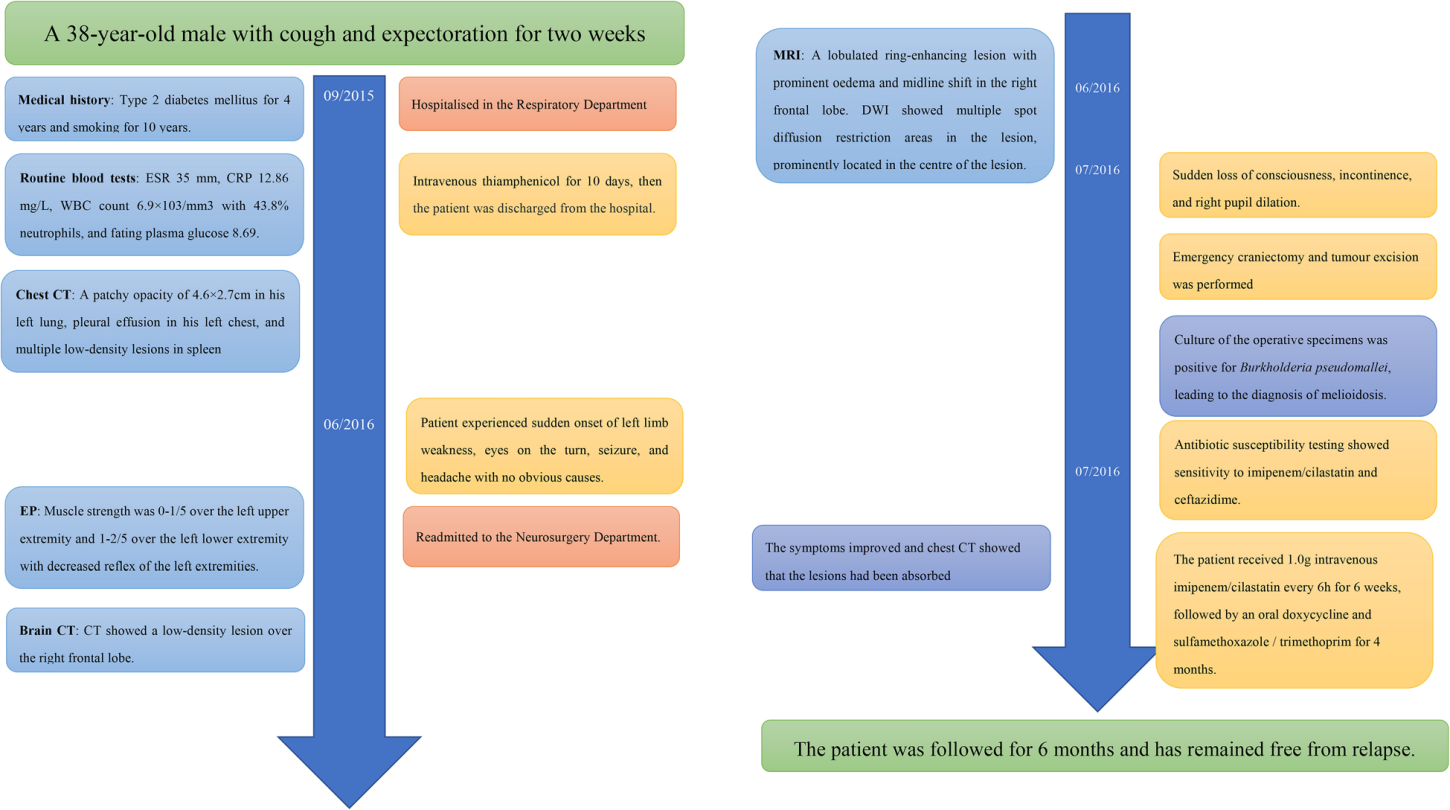
The timeline of the case. **a** 38-year-old male presented with cough and expectoration mixed with blood for two weeks and hospitalized into the department of respiratory on 09/2015. After 10 days of antibiotics treatment, his clinical symptoms improved and he was discharged from the hospital. But 8 months later (06/2016), the patient experienced sudden onset of left limb weakness and seizure and was re-admitted to the hospital. Brain CT and MRI showed a lesion over the right frontal lobe. Neuronavigation-guided open surgery was performed but failed to find a malignancy. **b** pseudomallei was cultured from the operative samples (07/2016). After 4 months of systemic and intraventricular antibiotic administration treatment, the patient achieved recovery (11/2016)

He received 1.0 g intravenous imipenem/cilastatin every 6 h for 6 weeks, and 0.1 g intravenous doxycycline twice a day for six weeks, followed by oral doxycycline and sulfamethoxazole/trimethoprim (SXT) for four months. His symptoms had improved and a chest CT showed that the lesions had been absorbed. He was discharged from the hospital on day 25 of treatment, and returned to his hometown for further antibiotic therapy (oral doxycycline and SXT for 4 months). Follow-up continued for 6 months. He remained free from relapse (Fig. 3).

## Discussion and conclusions

### Epidemiology

Hainan, a tropical region, offers favourable conditions for the spread of melioidosis. The vast majority of reports refer to sporadic cases with no easily discernible patterns. In 1997, approximately 60 cases from Hainan were reported, but there was no case of multi-systemic melioidosis with thorax, CNS, and spleen involvement [4, 5]. Occurrence of this disease is related with rainfall. The pathogens exist in the soil. During extreme weather, for example typhoon, the pathogens are aerosolised and inhaled. Therefore direct skin inoculation and inhalation are main invasion routes [6].

### Clinical features

The clinical manifestation of multi-systemic melioidosis varies depending on the organs involved. Pneumonia is the most common presentation of melioidosis (approximately 50%). Other forms of infection include prostatic abscess, parotitis, skin and soft tissue infections, and encephalomyelitis. CNS melioidosis has been increasingly reported in recent years [7], and neurological melioidosis has a high mortality rate of approximately 25% [8, 9]. It should require more attention.

Diabetes mellitus, chronic renal failure, malignant tumours, alcohol abuse, liver disease, steroid use, and lymphoid or myeloid disorders are the common predisposing factors to this disease. Such as in our case, many patients are young and have complications of diabetes mellitus [10, 11]. Diabetes mellitus might be a significant risk factor, especially for young people.

### Imaging features

Various radiological patterns of thoracic melioidosis have been recognised by clinicians. Acute disseminated infection is characterised by multiple lung nodules or multilobar consolidation with the subsequent formation and rupture of cavities, leading to pneumothorax or bronchopleural fistula. Subacute or chronic localised infection follows an insidious course and manifests as lobar, segmental consolidation, patchy opacities, or migrating opacities [12].

The radiological features in CNS melioidosis are different on stages, ranging from normal brain CT in the initial stages of cerebritis to typical macroabscess formation. MRI is more sensitive in the initial stages, with T2-weighted images detecting hyperintense changes in the brain parenchyma. The frontal lobes and brainstem are predisposing infection areas. Other radiological features include microabscesses, osteomyelitis, encephalitis, and myelitis. The typical imaging finding of CNS melioidosis is brain abscess. However, it is often difficult to differentiate melioidosis from brain malignancy using only conventional imaging. According to Liang et al. [13], DWI rises the confidence in distinguishing abscesses from cystic tumours. The criteria for pyogenic abscess are diffuse hyperintensity on DWI; the criteria for cystic tumour are hypointensity. The criteria result in 95% accuracy in diagnosis. Instead of typical diffusion restriction of the brain abscess, our patient shows sporophytic high signal on DWI. A possible explanation could be the early stage of the brain abscess with localised inflammation and abscess cavity formation. However, this case presented an early abscess that was misdiagnosed as a malignancy. Therefore, the possibility of abscess should be primarily considered when DWI shows diffuse hyperintensity or spot hyperintensity.

Melioidosis with multiple organs involved causes abscesses in those locations, such as liver, spleen, renal, and results in imaging manifestation of abscess on CT or MRI. Nevertheless, in our case, the organs were involved in sequence and the clinical symptoms were atypical, hampering the diagnosis [14].

### Treatment

A high index of suspicion for this infection, early diagnosis, and early initiation of high doses of appropriate antibiotics for a long duration are important because the mortality rates in infected individuals are otherwise very high [15]. Melioidosis can mimic tuberculosis in clinical presentation, imaging characteristics, and histopathology; therefore, patients with melioidosis might be inappropriately treated with antituberculous therapy. Treatment usually consists of intravenous antibiotics for 2–6 weeks, followed by oral maintenance therapy with cotrimoxazole, doxycycline, or quinolones for 4–8 months. The antibiotic medication used should have a good penetration into the brain tissue as well as activity against *B. pseudomallei*. The drug of choice for the treatment of melioidosis are ceftazidime or meropenem, alone or with SXT [16].

To conclude, this study reports on a 38-year-old male diabetic patient with multi-systemic melioidosis that affected the CNS, thorax, and spleen. It demonstrates some clinical, neurological, and radiological features and diagnostic challenges in multi-systemic melioidosis. CNS melioidosis in early stage manifest similar symptoms to malignancy or stroke. It might mislead to a false diagnosis. DWI can help differentiate abscesses from cystic tumours. It is extremely important to accurately diagnose before treatment is selected. Clinicians should stay alert of this disease in endemic areas in order to avoid misdiagnosis.

## Abbreviations

CNS: Central nervous system
CRP: C-reactive protein (CRP)
CT: Computed tomography
DWI: Diffusion weighed imaging
ESR: Erythrocyte sedimentation rate
GNB: Gram-negative bacterium
MRI: magnetic resonance imaging
SXT: Sulfamethoxazole / Trimethoprim
WBC: White blood cell
